# *Agrocybe aegerita* Polysaccharide Combined with *Bifidobacterium lactis* Bb-12 Attenuates Aging-Related Oxidative Stress and Restores Gut Microbiota

**DOI:** 10.3390/foods12244381

**Published:** 2023-12-05

**Authors:** Xiaoyan Liu, Yanyu Feng, Hongmin Zhen, Lina Zhao, Hongqiang Wu, Bin Liu, Guangsen Fan, Aijun Tong

**Affiliations:** 1 Beijing Engineering and Technology Research Center of Food Additives, School of Food and Health, Beijing Technology and Business University, Beijing 100048, China; liuxiaoyan@btbu.edu.cn (X.L.); zhenhongmin@btbu.edu.cn (H.Z.); 2 College of Food Science, Fujian Agriculture and Forestry University, Fuzhou 350002, China; fengyanyu1221@163.com (Y.F.); wuhq_2001@163.com (H.W.); 000q080003@fafu.edu.cn (B.L.); tajlove@163.com (A.T.); 3 China National Engineering Research Center of JUNCAO Technology, Fujian Agriculture and Forestry University, Fuzhou 350002, China; zln20002000@163.com

**Keywords:** *Agrocybe aegerita* polysaccharides, *Bifidobacterium lactis* Bb-12, antioxidant, anti-aging, gut microbiota

## Abstract

The objective of this study was to examine the impacts of the combing of *Agrocybe aegerita* polysaccharides (AAPS) with *Bifidobacterium lactis* Bb-12 (Bb-12) on antioxidant activity, anti-aging properties, and modulation of gut microbiota. The results demonstrated that the AAPS and Bb-12 complex significantly increased the average lifespan of male and female *Drosophila melanogaster* under natural aging conditions (*p* < 0.05), with an improvement of 8.42% and 9.79%, respectively. Additionally, the complex enhanced their climbing ability and increased antioxidant enzyme activity, protecting them from oxidative damage induced by H_2_O_2_. In D-galactose induced aging mice, the addition of AAPS and Bb-12 resulted in significantly increase in antioxidant enzyme activity, regulation of aging-related biomarker levels, changed gut microbiota diversity, restoration of microbial structure, and increased abundance of beneficial bacteria, particularly lactobacilli, in the intestines. These findings suggested that the complex of AAPS and Bb-12 had the potential to serve as a dietary supplement against organism aging and oxidative stress.

## 1. Introduction

Aging refers to a complex and irreversible natural process characterized by abnormal physiological degeneration, carcinogenesis, and even death, resulting from the decreased ability of an organism to maintain stability and respond to stress as it ages [1]. With the development of medical and technological advancements, the average lifespan of individuals has significantly increased, leading to the challenge of an aging population. The growing elderly population poses significant pressures on the economy, healthcare, and other sectors. Therefore, research related to aging has become a hot topic in the fields of food and medicine. Understanding the mechanisms of aging, establishing appropriate animal models to simulate the aging process, and finding safe and suitable natural products or interventions to intervene in the aging process are of great social significance in improving the quality of life for the elderly and achieving healthy aging [2].

In 1959, Denham Harman put forward the free radical theory on aging, which proposed that changes in cellular function during aging were attributed to the accumulation of reactive oxygen species (ROS) [3]. This accumulation causes oxidative damage to biomolecules and cells. Subsequently, this theory has been extensively investigated and acknowledged. Much research has demonstrated that ROS can impede cell growth, trigger cell death, and contribute to the aging process [4,5]. Oxidative stress-induced mitochondrial dysfunction, DNA rearrangements, genomic instability, protein dysfunction, as well as changes in metabolism and signaling pathways, have been found to exist in cells [6]. Therefore, the utilization of antioxidants is deemed a logical therapeutic approach for preventing and treating oxidative stress-related diseases and improving aging-related damage.

*Agrocybe aegerita*, commonly referred to as the chestnut mushroom, is a widely enjoyed edible mushroom known for its delightful aroma and flavor [7]. It boasts a wealth of nutrients and is abundant in several active constituents, such as polysaccharides, leucine and glutamate. One of its main active components, the *A. aegerita* polysaccharide (AAPS), has been found to have great potential in antioxidant and anti-aging functions in recent studies [8,9,10,11]. Studies by our research group have shown that AAPS exhibited potent antioxidant and anti-aging activity in vitro and in vivo, increasing cell viability, reducing mitochondrial membrane potential, prolonging the lifespan of flies, mitigating oxidative damage and restoring intestinal microbiota [12,13]. When combined with *Lactobacillus rhamnousus* GG, AAPS acted as a prebiotic and helped alleviate aging damage by regulating oxidative stress and gut microbiota [14]. Furthermore, AAPS had been shown to have multiple physiological functions, such as antioxidant, antitumor, anti-angiogenic, and thrombosis treatment properties [15,16,17]. Therefore, conducting in-depth research on the anti-aging function and mechanisms of AAPS combined with *Bifidobacterium lactis* Bb-12 (Bb-12) and expanding the development of anti-aging products based on AAPS as prebiotics has significant scientific significance.

The aim of this study was to determine whether the combination of AAPS and Bb-12 could have an anti-aging impact by reducing oxidative damage and regulating intestinal microbiota. To achieve this, we assessed the protective effects of natural aging and H_2_O_2_-induced oxidative stress on *D. melanogaster*, as well as their climbing ability. Additionally, we investigated the potential of AAPS combined with Bb-12 treatments to enhance antioxidant enzyme activities in *D. melanogaster* and mice, and to regulate aging biomarkers in mice. Furthermore, we analyzed the composition of the gut microbiota in mice in order to explore the relationship between the anti-aging and antioxidant function of AAPS combined with Bb-12 and its regulatory effect on intestinal microbiota.

## 2. Materials and Methods

### 2.1. Chemicals and Materials

*A. aegerita* (3MTJ-09106) was acquired from Gutian Tianxian Agricultural Products Co., Ltd. (Ningde, China). AAPS, a pure polysaccharide derived from *A. aegerita*, was prepared following established protocols [12]. The polysaccharide was extracted using ultrapure water and ultrasonic extraction, followed by concentration using a rotary evaporator. The concentrate was then mixed with ethanol and left overnight. The resulting precipitation was dissolved, and proteins were removed through neutral protease treatment. Subsequently, dialysis was performed using a 7 kDa molecular weight cutoff membrane for 48 h, followed by protein removal using the Sevage method. The crude polysaccharide was purified using DEAE cellulose-52 (Beijing Solarbio Science and Technology Co., Ltd., Beijing, China) and Sephadex G-100 (Beijing Solarbio Science and Technology Co., Ltd., Beijing, China). The resulting polysaccharide solution was collected for further analysis.

The Bb-12 strain was provided by the Food Biotechnology Laboratory of Fujian Agriculture and Forestry University (Fuzhou, China). Commercial kits for assessing the levels of total superoxide dismutase (T-SOD), catalase (CAT), glutathione peroxidase (GSH-Px), malondialdehyde (MDA), inducible nitric oxide synthase (iNOS), and total protein in mice were obtained from Nanjing Jiancheng Biochemical Co., Ltd. (Nanjing, China). Kits for measuring advanced glycation end products (AGEs) in mice and antioxidant activity in flies were obtained from Lerang Biotechnology Co., Ltd. (Fuzhou, China). PCR-ELISA kits for telomerase analysis were sourced from Runwell-tac Co., Ltd. (Shanghai, China). All other chemicals and reagents used were of analytical grade and commercially available.

### 2.2. Fly Strains and Diet

Wild-type *D. melanogaster* (Oregon) fly strains were obtained from the Food Biotechnology Laboratory of Fujian Agriculture and Forestry University. The flies were reared in an incubator under controlled conditions of (25 ± 1) °C, 50–60% humidity, and a 12-h light/dark cycle. The basic culture medium was prepared using the following ingredients: 72 g of corn flour, 72 g of anhydrous glucose, 6 g of agar, 10 g of dry yeast, and 6 mL of propionic acid. Anhydrous glucose and agar were gradually added to 700 mL of water, heated, and continuously stirred until fully dissolved. The mixture was then combined with the evenly mixed corn paste, brought to a boil, and cooled to 50 °C. Dry yeast powder and propionic acid were added and stirred thoroughly. The resulting mixture was poured into sterile culture tubes, with each tube having a height of approximately 2.0–3.0 cm. The diets were prepared every three days.

For the experimental group, the culture medium was prepared by adding 9 mg/mL of AAPS, 10^9^ CFU/mL of Bb-12, or a combination of both to the basic culture medium during the final preparation step.

### 2.3. Lifespan Assay

A combined total of 800 male and female flies, which had not engaged in mating within 8 h of eclosion, were gathered and then randomly segregated into four groups: a control group and three experimental groups. Each group consisted of 100 males and 100 females. The normal control group (NC) was fed with the basic culture medium, while the experimental groups were fed with the following experimental medium: 9 mg/mL AAPS (AA), 10^9^ CFU/mL Bb-12 (BB), and a combination of 9 mg/mL AAPS and 10^9^ CFU/mL Bb-12 (AB), respectively. The number of dead flies was recorded daily at a fixed time. The mean lifespan, maximum lifespan and median survival of the flies were statistically analyzed and plotted on a survival curve. The mean lifespan of the last 10 flies in each group was considered as the maximum lifespan.

### 2.4. Hydrogen Peroxide (H_2_O_2_) Challenge Assay

To induce oxidative stress, flies were exposed to H_2_O_2_. After 20 days of being fed with either the basic medium or one of the experimental media (AA, BB, or AB), a total of 800 male and female flies were gathered and then randomly distributed into four groups, each consisting of 200 flies (half male and half female). These flies were then transferred into culture tubes containing filter paper soaked with 200 µL of a 30% H_2_O_2_ solution in a 6% glucose solution. The volume of solution added was adequate to saturate the filter paper without any excess liquid. The number of deceased flies was recorded every three hours until all flies had perished. Subsequently, the mean lifespan, maximum lifespan, and median survival of the flies were subjected to statistical analysis and plotted on a survival curve.

### 2.5. Climbing Ability Assay

A total of 240 male and female flies were randomly assigned to four groups, each group containing 60 flies (half male and half female). The groups were fed different diets, including a control group (NC) and three experimental groups (AA, BB, and AB). The flies were placed in an incubator at 25 °C for either 10 or 30 days. After the incubation period, the flies were subjected to a climbing assay under reversed gravity conditions. The assay involved transferring the flies to dry flat-bottomed tubes and tapping the tubes to make the flies fall to the bottom. The count of flies that reached a pre-established 8 cm mark within a span of 10 s was documented. The process was repeated five times with intervals of at least 1 min between each measurement. A higher climbing index indicated stronger climbing ability against gravity. The fly climbing ability index was calculated accordingly.

Climbing ability index = (number of flies successfully climbing to 8 cm/total number of flies).

### 2.6. Animals and Treatment

Six-week-old male Kunming mice were procured from Wu’s Laboratory Animal Co. Ltd. in Fuzhou, China. The mice were handled and cared for in accordance with the Guidelines for the Care and Use of Laboratory Animals. All animal experiments were conducted following the ethical guidelines and procedures approved by the Academic Committee of Fujian Agriculture and Forestry University (ACFAFU32033). The mice were housed at a temperature of 22 ± 2 °C and a 12-h light-dark cycle, with ad libitum access to food and water. After a one-week adaptation period, the mice were randomly divided into five groups, each comprising 10 mice, and treated for 8 consecutive weeks. The groups and treatments were as follows: the normal control group (NC), the aging model control group (MC), the group orally administered AAPS at a dose of 400 mg/kg·bw/day (AA), the group orally administered Bb-12 at a concentration of 10^9^ CFU/mL/day (BB), and the group orally administered a combination of AAPS (400 mg/kg·bw/day) and Bb-12 (10^9^ CFU/mL/day) (AB). With the exception of the NC group, all other groups were subcutaneously injected with D-galactose dissolved in normal saline (0.9%, *w*/*v*) at a dose of 120 mg/kg·bw once daily for 8 weeks to induce the aging model. At the end of the experiment, all animals were euthanized, and their serum, brain, and cecal contents were collected for future use.

### 2.7. Biochemical Analysis

Following a 30-day treatment with various media, flies from each group were collected, euthanized by freezing, and homogenized with normal saline. The homogenate was then centrifuged, and the supernatant was utilized for protein content and antioxidant activity measurements (T-SOD activity, CAT activity, GSH-Px activity, and MDA content). Blood samples were centrifuged to obtain the serum supernatant. The brain tissues were homogenized and centrifuged, and the resulting supernatant was collected. The levels of aging biomarkers (iNOS, telomerase, and AGEs) and antioxidative capacities (T-SOD activity, CAT activity, GSH-Px activity, and MDA content) were determined using commercially available kits according to the manufacturer’s protocols. Protein determination in the sample was conducted using the Bradford method. Protein molecules contain -NH^3+^ groups, and when the brown-red Coomassie Brilliant Blue G-250 dye was added to the sample, the anions on the dye bound to the protein -NH^3+^ groups, causing the solution to turn blue. The protein content was calculated by measuring the absorbance.

### 2.8. Intestinal Microbiota Analysis

DNA was isolated from the cecal content of mice using the MoBio Power DNA extraction kit. High-quality DNA samples were chosen for library construction and sequencing. The samples were diluted to a concentration of 1 ng/μL with sterile water. The V3-V4 region of the 16S rRNA gene was amplified using general primers and Trans Start Fast pfu DNA polymerase. Paired-end amplicon sequencing of the amplified DNA templates was performed using the Illumina MiSeq-PE250 system. The resulting sequencing data were analyzed using the RStudio (Auckland, New Zealand) and Majorbio I-Sanger Cloud Platform (Shanghai, China). Species information and abundance distribution were obtained based on the operational taxonomic unit (OTU) clustering results. Alpha diversity analysis of the OTUs was conducted to evaluate species abundance and evenness within samples. Multiple sequence alignment of the OTUs allowed for further analysis of community structure differences among different groups. Beta diversity analysis was carried out to assess the microbial community composition of different samples, and the results were visualized using PCA plots. Correlation analysis with environmental factors was performed to identify significant factors influencing inter-group community variations.

### 2.9. Data Analysis

The results were expressed as mean ± standard error of mean (Mean ± SEM). Data visualization and statistical analysis were performed using GraphPad Prism 9.5 software. Survival analysis was carried out using the log-rank test to assess differences in life expectancy significance. The normal distribution of the data was evaluated using the D’Agostino and Pearson test, and the parametric Student’s *t*-test was conducted accordingly. A *p*-value less than 0.05 or 0.01 was considered statistically significant for mean differences.

## 3. Results

### 3.1. Effect on the Longevity of D. melanogaster

**Figure 1:**
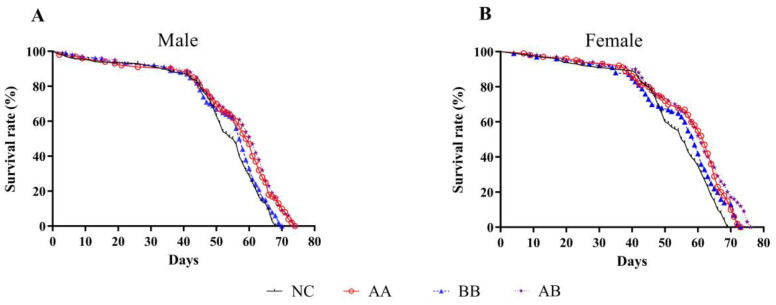
Effects of AAPS and Bb-12 on the lifespan of *D. melanogaster*. (**A**) Male. (**B**) Female. AA, 9 mg/mL AAPS; BB, 10^9^ CFU/mL Bb-12; AB, a combination of 9 mg/mL AAPS and 10^9^ CFU/mL Bb-12; (n = 100 per group).

*D. melanogaster*, commonly known as the fruit fly, is frequently employed to investigate anti-aging activity and mechanisms in animals owing to its brief lifespan, rapid reproductive rate, uncomplicated feeding conditions, and metabolic pathways and aging genes that are similar to those in humans [18]. When comparing the impacts of dietary interventions, it was discovered that the median survival time, mean lifespan, and maximum lifespan of both male and female flies in the AB group were significantly greater than those in the NC group (*p* < 0.05) (Table 1). The mean lifespan of male and female flies in the AB group was extended by 8.42% and 9.79%, respectively. The median survival time of male flies in the AA, BB, and AB groups was extended by 4, 2, and 5.5 days, and the median survival time of female flies was extended by 6.5, 3.5, and 5.5 days, respectively. All three groups significantly extended the maximum lifespan of flies, with the AB group showing the best extension effect. By comparing male and female groups, it was observed that the mean lifespan, maximum lifespan, and median survival time of female flies were higher than those of male flies in almost all cases, which might be due to genetic differences between female and male flies and their different sensitivities to the tested drugs. The Kaplan-Meier survival curves (Figure 1A,B) showed during the early growth stage of flies (0–40 days) that there was no significant difference in survival rate between the NC group and the experimental groups. However, the experimental groups had a higher survival rate than the NC group after 50 days, with the AB group showing the most pronounced longevity effect.

### 3.2. Effect on H_2_O_2_-Induced Intensive Oxidative Stress in D. melanogaster

**Figure 2:**
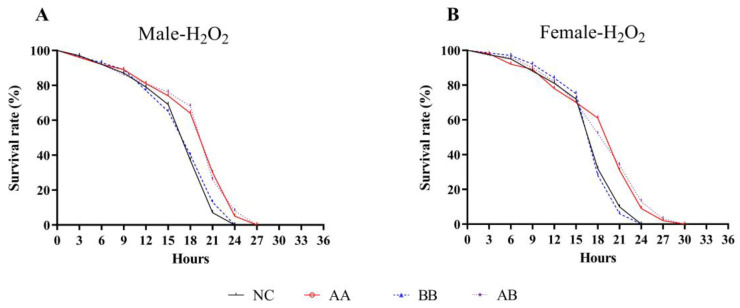
Effects of AAPS and Bb-12 on the lifespan of H_2_O_2_ treated *D. melanogaster* .(**A**) Male. (**B**) Female. AA, 9 mg/mL AAPS; BB, 10^9^ CFU/mL Bb-12; AB, a combination of 9 mg/mL AAPS and 10^9^ CFU/mL Bb-12; (n = 100 per group).

Diet interventions that extend lifespan usually helped enhance the organism’s resistance to various forms of physiological stress. In order to determine whether the mechanism by which AAPS combined with Bb-12 extends lifespan was related to improved stress resistance, the impacts of this combination on the lifespan under oxidative stress conditions were evaluated. Results showed that the median survival time, mean lifespan, and maximum lifespan of both male and female flies in the AA and AB groups were significantly higher than those in the NC group (*p* < 0.05) (Table 2). Specifically, the mean lifespan of male and female flies in the AB group was extended by 12.15% and 10.21%, respectively (*p* < 0.05). While the mean lifespan of the BB group did not change significantly, the maximum lifespan was extended (*p* < 0.05). The median survival time of male and female flies in the AA and AB groups was extended by 3 h compared to the NC group. The Kaplan-Meier survival curves in Figure 2A,B showed that the combination of AAPS and Bb-12 shifted the survival curve to the right, indicating an extension of the survival time of flies under oxidative stress conditions. These results suggested that AAPS combined with Bb-12 might play a beneficial role in intervening in the antioxidant defense mechanism of flies, thereby extending their lifespan.

### 3.3. Effect on the Climbing Ability of D. melanogaster

**Figure 3:**
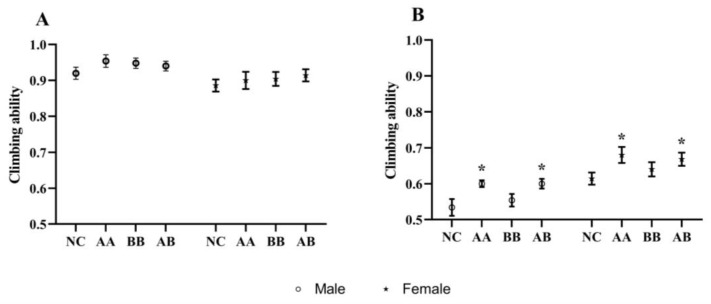
Effects of AAPS and Bb-12 on the climbing ability of *D. melanogaster*. (**A**) Male. (**B**) Female. AA, 9 mg/mL AAPS; BB, 10^9^ CFU/mL Bb-12; AB, a combination of 9 mg/mL AAPS and 10^9^ CFU/mL Bb-12; (n = 30 per group). Data are presented as the means ± SEM. * *p* < 0.05 vs. NC group.

Behavioral decline was a common characteristic of aging in animals, and it could be observed in the climbing assay designed for flies based on their locomotor abilities. The climbing index was used to evaluate their locomotor ability, and it typically decreases with age [19]. The average climbing index of the experimental groups on the 10th day was slightly higher than that of the NC group, although the difference was not significant (Figure 3A). However, on the 30th day, both the AA and AB groups exhibited significantly higher climbing indices compared to the NC group (Figure 3B). Two-way ANOVA-Tukey analysis showed a significant difference in climbing ability between male and female fruit flies on the tenth and thirtieth days (*p* < 0.01). Furthermore, significant differences in climbing ability were also observed on the 30th day due to different dietary interventions (*p* < 0.01). These findings suggested that the combination of AAPS and Bb-12 could effectively enhance the climbing ability of 30-day-old flies, indicating a potential positive impact of this combination on the locomotor ability of naturally aging flies.

### 3.4. Effect on the Level of Antioxidant Activities in D. melanogaster

**Figure 4:**
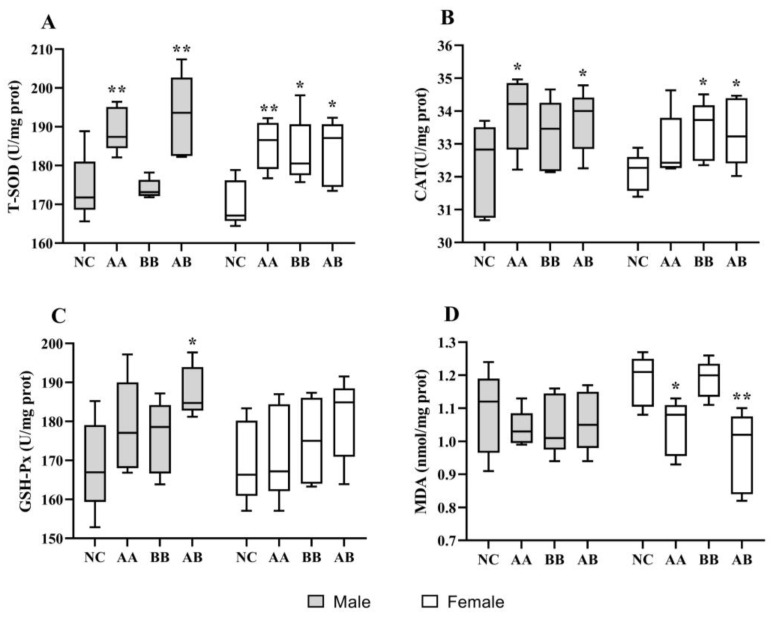
Effects of AAPS and Bb-12 on the antioxidant capacity of *D. melanogaster*. (**A**) T-SOD. (**B**) CAT. (**C**) GSH-Px. (**D**) MDA. AA, 9 mg/mL AAPS; BB, 10^9^ CFU/mL Bb-12; AB, a combination of 9 mg/mL AAPS and 10^9^ CFU/mL Bb-12; (n = 100 per group). Data are presented as the means ± SEM. * *p* < 0.05 vs. NC group. ** *p* < 0.01 vs. NC group.

Oxidative stress occurs when the antioxidant system was unable to effectively clear free radicals and toxic metabolites due to various internal and external factors. In the H_2_O_2_-induced experiment, it was observed that treatment with AAPS combined with Bb-12 extended the survival time of flies under oxidative stress conditions, indicating a beneficial role of intervening in the antioxidant defense mechanism of flies. After 30 days of AAPS combined with Bb-12 treatment, significant improvements in antioxidant activities were observed (Figure 4). Both male and female flies in the AA and AB groups exhibited significantly higher T-SOD activity compared to the NC group, and female flies in the BB group also showed a significant improvement (*p* < 0.05) (Figure 4A). As shown in Figure 4B, although the average CAT levels in each experimental group were higher than the NC group, only the CAT activity in male flies of the AA and AB groups and in female flies of the BB and AB groups was significantly enhanced (*p* < 0.05). While the mean level of GSH-Px was higher in the treatment groups compared to the NC group, statistically significant improvement was observed only in the AB group of male flies, not in the other groups (Figure 4C). Figure 4D demonstrates that the MDA levels in female flies of the AA and AB groups were significantly lower than the NC group, indicating that AAPS combined with Bb-12 had a delaying effect on the increase of MDA levels in the body. When comparing male and female flies, it was observed that the changes in MDA levels induced by AAPS combined with Bb-12 were more pronounced in female flies. This could be attributed to the larger body size and higher lipid content in female flies, leading to higher levels of lipid peroxidation and more significant changes. Compared to the individual interventions of AAPS or Bb-12, the combined intervention demonstrated the most effective enhancement in antioxidant activity. The 2-way ANOVA-Tukey analysis showed that different dietary interventions resulted in significant differences in antioxidant capacity across all antioxidant indicators (*p* < 0.05). However, gender did not have a significant impact on the antioxidant indicators (*p* > 0.05). This could happen by regulating the antioxidant defense system, decreasing the buildup of oxidative damage in the body, and thereby prolonging the lifespan of flies.

### 3.5. Effect on the Level of Antioxidant Activities and Aging Biomarkers in Mice

Table 3 presents the impact of AAPS combined with Bb-12 on the levels of aging biomarkers in mice. The MC group displayed a significant decrease in T-SOD, GSH-Px, and CAT activities in both serum and brain tissue compared to the NC group (*p* < 0.01). However, all diet intervention groups exhibited a significant increase in T-SOD activity in both serum and brain tissue compared to the MC group (*p* < 0.01). The AA and AB groups showed higher CAT and GSH-Px activities in both serum and brain tissue compared to the MC group (*p* < 0.05), while the brain tissue of the BB group did not show significant changes (*p* > 0.05). The MDA levels in both serum and brain tissue significantly decreased in all diet intervention groups (*p* < 0.05). These findings indicated that AAPS combined with Bb-12 enhanced antioxidant activity in mice.

Regarding the levels of aging biomarkers, Table 3 demonstrates that the MC group exhibited significant alterations in all aging biomarkers compared to the NC group (*p* < 0.01). All diet intervention groups showed a significant decrease in iNOS compared to the MC group (*p* < 0.01). The AB group also displayed a significant decrease in AGEs (*p* < 0.05), while the changes in other groups did not reach statistical significance (*p* > 0.05). The changes in telomerase activity were not significant across all diet intervention groups (*p* > 0.05). These results suggested that AAPS combined with Bb-12 influenced the levels of certain aging biomarkers in mice, but the activity of telomerase might be influenced by multiple factors. Therefore, while the mean values of telomerase showed improvement, they did not exhibit statistical significance. The 2-way ANOVA-Tukey analysis showed that different dietary interventions did not result in significant differences in the anti-aging and antioxidant indicators in mice. However, the Student’s *t*-test analysis yielded slightly different results. This may be due to differences in statistical analysis methods, as the Student’s *t*-test focuses more on differences between the two indicators, while the Two-way ANOVA-Tukey analysis places more emphasis on overall differences. This also proved from another perspective that dietary intervention can only improve the antioxidant activity and aging indicators in animals to a certain extent. Dietary intervention should be combined with other methods to make the body healthier.

### 3.6. Effect on the Gut Microbiota Composition of Mice

**Figure 5:**
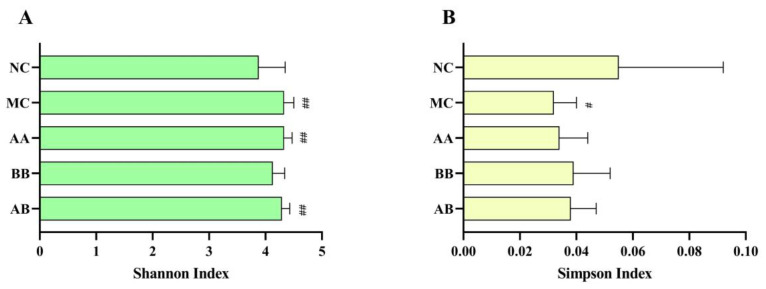
Effects of AAPS and Bb-12 on Alpha diversity analysis indexes among groups. (**A**) Shannon index. (**B**) Simpson index. (n = 10 per group). Data are presented as the means ± SEM. ^#^ *p* < 0.1 vs. NC group. ^##^ *p* < 0.01 vs. NC group.

The assessment of species abundance and evenness in the gut microbiota included two different alpha diversity indices (Shannon and Simpson), which reflected the complexity of the sample community. The results from Figure 5 revealed significant differences (*p* < 0.1) in the two alpha diversity indices between the MC group and the NC group. In the diet intervention groups, the Simpson index was significantly higher in the BB and AB groups compared to the MC group (*p* < 0.001). This indicated that the diet intervention of AAPS and Bb-12 had a moderating effect on gut microbiota.

**Figure 6:**
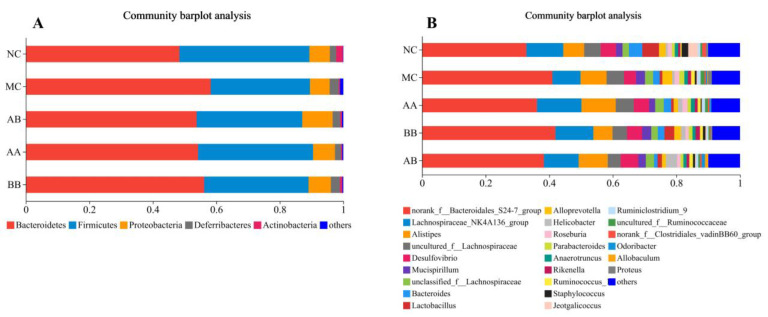
Effects of AAPS and Bb-12 on relative abundance of the topmost dominant intestinal bacterial among groups. (**A**) Level of phylum classification. (**B**) Level of genus classification. (n = 10 per group).

At the phylum level, the top five species with the highest abundance in mice intestine were *Bacteroidetes*, *Firmicutes*, *Proteobacteria*, *Deferribacteres*, and *Actinobacteria* (Figure 6A). Compared to the NC group, the MC group exhibited a decrease in abundance of *Firmicutes* and *Actinobacteria*, while the abundance of *Bacteroidetes* and *Deferribacteres* increased. The diet intervention effectively reversed these changes. Figure 6B indicates that diet intervention had a significant impact on the genus-level composition of the gut microbiota in aging mice. The abundance of *Lactobacilli* in the NC group, BB group, and AB group was significantly higher than that in the MC group.

**Figure 7:**
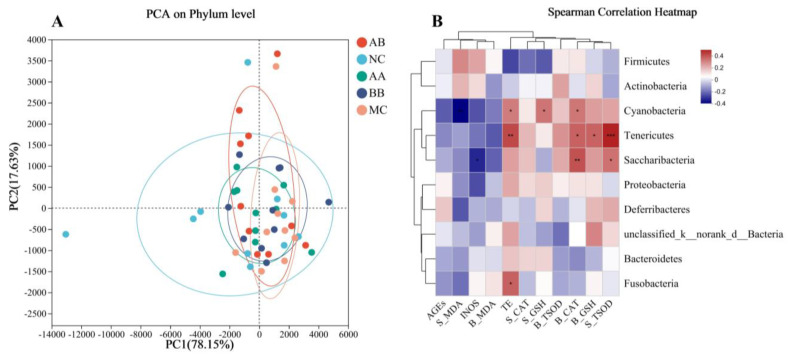
Beta diversity analysis of gut microbiota and correlation analysis between the relative abundance of gut microbiota and biomarkers. (**A**) Principal component analysis (PCA) on phylum level based on Bray-Curtis metrics. Points of different colors or shapes represent samples from different groups. The closer two sample points are, the more similar the compositions of the two sample species. Using circles to enclose most of scatter plots corresponding to samples of the same group, the size of the circle indicated the degree of dispersion of the samples in the same group. (**B**) Spearman correlation heatmap analysis between the relative abundance of gut microbiota and the biomarkers, * 0.01 < *p* ≤ 0.05, ** 0.001 < *p* ≤ 0.01, *** *p* ≤ 0.001.

The similarities and differences in microbial evolution among various groups were visualized using PCoA analysis. As shown in Figure 7A, the sample area range of the NC group was larger and significantly different from the other groups, showing that the abundance and diversity of the gut microbiota in mice underwent significant changes under the influence of D-lactose injection. Although there was some overlap between the samples from the diet intervention group and the MC group, the range was significantly expanded, indicating that the diet intervention with AAPS combined with Bb-12 partially restored the gut microbiota.

To study the correlation between changes in gut microbiota and physiological and biochemical indicators in mice after diet intervention, a Spearman correlation heatmap was created (Figure 7B). The relative abundance of *Cyanobacteria* was significantly positively correlated with the level of telomerase in the brain, GSH-Px in serum, and CAT in the brain, and significantly negatively correlated with the level of MDA in serum. The relative abundance of *Tenericutes* was significantly positively correlated with the level of T-SOD in serum, GSH-Px in the brain, CAT in the brain, and telomerase in the brain. The relative abundance of *Saccharibacteria* was significantly positively correlated with the level of T-SOD in serum and CAT in the brain, and significantly negatively correlated with the level of iNOS in the brain. The relative abundance of *Fusobacteria* was significantly positively correlated with the level of telomerase in the brain.

## 4. Discussion

Edible plants are known to contain various natural bioactive compounds, which serve as the material basis for their biological activity and health benefits. Fungal polysaccharides are derived from edible fungi. β-glucans, the primary polysaccharides in most mushrooms, including AAPS, are resistant to hydrolysis by mammalian digestive enzymes [20] Intestinal microorganisms contain a variety of carbohydrate-active enzymes that degrade polysaccharides and oligosaccharides, fermenting them to produce short-chain fatty acids (SCFAs) [21]. Wu et al. reported that the SCFA content in the cecum of mice injected with D-galactose was lower than that of the healthy group [14]. After AAPS and *Lactobacillus rhamnosus* GG dietary intervention, the content of SCFA slightly recovered but there was no significant improvement. Fungal polysaccharides exert diverse regulatory and protective effects on the gut microbiota, enhancing intestinal integrity, reducing mucosal damage, promoting the growth of beneficial bacteria, and increasing SCFA production [22,23].

Age is closely linked to the composition of the gut microbiota, and elderly individuals typically exhibit increased numbers of harmful microorganisms in their gut microbiota [24]. Studies have shown that improving the composition, metabolic products, and functions of the gut microbiota through the supplementation of probiotics and prebiotics could enhance the health status of elderly individuals and alleviate age-related damage [25,26,27]. Synbiotics, which combine probiotics and prebiotics, are a type of bioproduct that can promote the colonization, growth, and reproduction of both exogenous and endogenous bacteria in the gut, thereby exerting dual effects [28,29]. This leads to increased survival rates of beneficial exogenous bacteria, which, in turn, inhibits the growth of intestinal pathogens and improves the structure of the gut microbiota, ultimately enhancing the body’s immune system. Polysaccharides serve as excellent prebiotics for probiotics, and the two can complement each other and work synergistically. AAPS is extracted from *Agrocybe aegerita* mushrooms. Research on AAPS suggests that it might possess better antioxidant and anti-aging activities when used as a prebiotic [14]. The results of this study demonstrated that the combined use of AAPS and Bb-12 could prolong the lifespan of flies, effectively improve the antioxidant levels in flies and mice, and enhance the gut microbiota of aging mice.

Aging is a natural process marked by a decrease in bodily functions, disturbance of the internal environment, diminished stress resistance, and irreversible degenerative alterations in the structure and function of organisms as time passes. Lifespan is the primary indicator for evaluating the impact of dietary interventions on the aging rate of flies. In this study, AAPS, Bb-12, and their combination were added to the culture medium used for feeding flies. By measuring the average, median, and maximum lifespan, we found that the combined use of AAPS and Bb-12 had the optimal longevity effect (Figure 1). During the short lifespan of flies, their physical abilities, such as flight and climbing, gradually decline with age. Previous studies had shown that the time required for a 45-day-old fly to climb 17.5 cm was twice that of a 5-day-old fly [30]. Furthermore, within 5 to 50 days after eclosion, flies experienced a 40–60% decrease in climbing speed [31]. Measuring the climbing ability of flies reflected the integrated function of neurons and muscles [32]. Results in this study showed that, although the combination of AAPS and Bb-12 had no significant improvement on the climbing ability of 10-day-old flies (*p* > 0.05), it significantly enhanced the climbing ability of 30-day-old flies, which might be related to the antioxidant and neuroprotective effects of AAPS combined with Bb-12 (Figure 3).

As molecular biology and life sciences have advanced, numerous theories on the mechanisms of aging have been proposed by scholars, with the free radical theory being one of the most important representative theories [33,34]. Free radicals are formed by the breaking of covalent bonds in elements or compounds. Oxygen free radicals are the primary free radicals in organisms, and the body normally maintains a dynamic equilibrium between the production and elimination of free radicals [35]. When this dynamic balance is disrupted, cellular functions are altered or even lost, thereby accelerating the aging process. D-Galactose is an isomer of glucose that has reducing properties and can react with glucose to produce lactate, regulating energy conversion and substance metabolism. For healthy adults, the recommended maximum daily dose of galactose is 50 g, and within approximately 8 h after ingestion, most galactose can be metabolized and excreted from the body [36]. However, in cases of excessive intake or abnormal body conditions, under the catalysis of galactose oxidase, it can be converted into aldehydes and hydrogen peroxide, causing oxidative damage [37]. Inducing aging with D-galactose involves continuously injecting animals with D-galactose for a certain period of time, increasing the concentration of D-galactose in the body, causing the accumulation of D-galactose alcohol, disrupting osmotic pressure, disrupting metabolism, depleting antioxidant substances, damaging cells, and accelerating aging. Many studies have confirmed that the D-galactose-induced aging mouse model could be used for the activity evaluation of anti-aging drugs [38,39].

It is widely believed that longevity requires the enhancement of the body’s protection against oxidative stress and its byproducts. Endogenous antioxidant cellular defenses include enzymes and non-enzymatic molecules distributed in the cytoplasm and organelles. In this study, the combination of AAPS and Bb-12 not only extended the lifespan of flies but also enhanced their resistance to oxidative damage. Under the H_2_O_2_-induced oxidative stress, the combination of AAPS and Bb-12 resulted in longer survival time (Figure 2). Furthermore, AAPS combined with Bb-12 upregulated the levels of T-SOD, CAT, and GSH-Px in flies, which helped enhance the body’s antioxidant system (Figure 4). This was consistent with the conclusions obtained in our D-galactose-induced aging mice experiment (Table 3). The oxidative system in aging mice was significantly improved under diet intervention. The continuous regulation of the production, localization, and inactivation of ROS was maintained by the delicate balance between the oxidation and antioxidant mechanisms. Endogenous antioxidants and exogenous antioxidants, together, constituting an indispensable detoxification system for ROS [40]. However, when various factors both inside and outside the body prevent the antioxidant system from clearing free radicals and toxic metabolites, oxidative stress occurs. The body’s ability to remove free radicals is an important factor determining the speed of aging. SOD is a metal enzyme that initiates the transformation of superoxide radicals into hydrogen peroxide and oxygen. It serves as the primary defense against oxidative damage induced by ROS [41]. Its activity is closely related to aging and gradually decreases with age. GSH-Px often works in conjunction with SOD to maintain the body’s internal environment. GSH-Px is susceptible to inactivation by superoxide radicals, but the metal elements in SOD can react with these radicals to protect GSH-Px from oxidation. SOD is susceptible to inactivation by hydrogen peroxide, but GSH-Px can catalyze the decomposition of hydrogen peroxide, thereby protecting SOD. The decline in GSH-Px activity weakens the ability to remove free radicals and increases lipid peroxidation. This result is consistent with the “oxidative excess” theory of aging caused by D-galactose, confirming that the imbalance in the metabolism of ROS leads to aging. CAT acts similarly to SOD and can eliminate free radicals in the body, thereby reducing the production of peroxides. The formation of MDA can be induced by non-enzymatic or enzymatic reactions of ROS [42]. The former can induce lipid peroxidation reactions in cell membranes, leading to the accumulation of lipid peroxides, which can exacerbate oxidative damage to cell membranes. This study found that the antioxidant level of the MC group of mice was significantly lower than that of the NC group, indicating that the MC group experienced severe oxidative damage under the action of D-galactose (Table 3). Compared to the MC group, the combination of AAPS and Bb-12 exhibited strong antioxidant activity. The T-SOD, CAT, and GSH-Px levels in the AA and AB groups were significantly higher than those in the MC group, and MDA was significantly reduced. This may be due to the upregulation of mRNA expression levels related to antioxidant enzymes, which eliminates the production of lipid peroxides by inhibiting oxygen free radicals and enhances the activity of antioxidant enzymes, thereby regulating the antioxidant defense system, reducing the accumulation of oxidative damage in the body, and delaying aging.

This study found that the complex of AAPS and BB12 had positive effects on aging biomarkers in addition to enhancing the antioxidant enzyme activity in the body. Compared to the MC group, the AA and AB groups showed significant reductions in AGEs, iNOS, and lactic acid (*p* < 0.01) (Table 3). AGEs accumulate continuously in the serum or tissues of humans and animals as they age. Under physiological conditions, AGEs that are not easily degraded can bind to tissues and cells in the body and activate multiple signaling pathways. This induces oxidative damage, mitochondrial dysfunction, chronic inflammation, protein denaturation, and cell apoptosis, affecting the normal structure and function of tissue cells and disrupting the homeostatic balance of the internal environment. Ultimately, this leads to degenerative diseases in organs such as the liver, brain, and kidneys [43,44]. Under normal physiological conditions, only a small amount of iNOS is expressed in tissue cells. However, when the internal environment is subjected to oxidative stress and the induction of inflammatory reactive factors, its activity is significantly increased. The complex of AAPS and BB12 significantly reduced the levels of AGEs, iNOS, and lactic acid in mice modeled with D-galactose (Table 3). This indicated that the complex of AAPS and BB12 has a protective effect on the brain tissue of mice and alleviates oxidative stress-induced damage. Telomerase is a reverse transcriptase that carries repetitive sequences containing nucleotides to maintain telomere length [45]. Each time a cell divides, the telomere length shortens. When it reaches a certain degree of shortening, the cell no longer divides and undergoes aging and apoptosis. Although the dietary intervention of AAPS and Bb-12 could increase the levels of telomerase in mice to some extent, there was no significant difference compared to the MC group. This might be due to the influence of multiple factors on telomerase, making it difficult to achieve a significant change.

The human gut is a complex ecosystem that contains approximately 10–100 trillion organisms, including bacteria, eukaryotes, and archaea, collectively known as the gut microbiota. The composition and structure of the gut microbiota are closely related to age [46,47,48]. Gut diversity represents the species diversity and quantity in the gut microbiota. In this study, the results of alpha diversity analysis (Figure 5) showed that, in the D-galactose-induced aging mouse model, compared to the NC group, Shannon and Simpson index were significantly changed, while the dietary intervention regulated gut microbiota diversity compared to the aging group. Therefore, regulating gut microbiota diversity can promote the body’s antioxidant capacity, but further research is needed to support this mechanism. The gut microbiota structure in elderly individuals is significantly different from that of young people. With age, the abundance of *Firmicutes* decreases in elderly subjects [49,50]. These findings were consistent with our research results. Results in this study showed that dietary intervention with AAPS combined with Bb-12 effectively promoted the increase in the abundance of *Firmicutes*, *Proteobacteria* and *Actinobacteria* in the gut, while reducing *Bacteroidetes* and *Deferribacteres* (Figure 6A).

By promoting the proliferation of beneficial bacteria and the production of SCFAs, fungal polysaccharides can lower the pH in the intestines, inhibit the activation of the transcription factor NF-kB and immune inflammatory responses, and thereby suppress pathogenic bacteria while reducing the proportion of harmful intestinal microorganisms [22]. Members of the *Lactobacillus* family are recognized as beneficial host-associated groups in the human and animal microbiota. Changes in the abundance of gut *Lactobacilli* have been associated with oxidative stress and various diseases, including liver fibrosis, adipose tissue homeostasis and hypertension [51,52,53,54]. Supplementing or adjusting *Lactobacilli* can be used as a therapeutic intervention for human health and disease [55]. Kong. [56] suggested that *Lactobacilli* regulated the body’s antioxidant stress through the Keap1/Nrf2/ARE signaling pathway. Finamore et al. [57] found that *Lactobacillus casei* reduced oxidative stress-induced membrane barrier damage. Li et al. [58] demonstrated that *Lactobacilli* had good free radical scavenging and anti-aging abilities both in vitro and in vivo. In this study, adding Bb-12 and a mixture of AAPS and Bb-12 to the diet significantly increased the abundance of *Lactobacilli* in the gut microbiota of aging mice (Figure 6B). Based on the results of this experiment, dietary intervention with AAPS and Bb-12 effectively reduced the abundance of intestinal inflammation-related bacteria such as uncultured*_f__Lachnospiraceae*, *Anaerotruncus*, uncultured*_f__Ruminococcaceae*, and *Proteus* (Figure 6B). Uncultured*_f__Lachnospiraceae* was positively correlated with dextran sodium sulfate-induced ulcerative colitis [59]. *Anaerotruncus* was believed to be associated with cognitive impairment in peritoneal dialysis patients [60]. *Proteus* had many potential virulence factors related to the gastrointestinal pathogenicity and the ability to acquire antibiotic resistance. *Proteus* was a low-abundance symbiont in the intestinal tract with significant pathogenic potential [61]. Therefore, AAPS combined with Bb-12 selectively promoted the proliferation of beneficial bacteria in the intestines, suppresses the abundance of harmful bacteria in the intestines, and optimizes the intestinal microecological environment. In recent published literature, in HFD-induced animal models, *Sarcodon aspratus* polysaccharides were found to increase the abundance of *Lactobacillus* and *Bacteroides* [62]. Similarly, oral *Ganoderma lucidum* polysaccharides had been reported to alleviate gut microbiota imbalance by reducing the abundance of *Proteus* [63] Furthermore, seleno-lentinan had been shown to enhance the relative abundance of beneficial bacteria, such as *Lactobacillus*, and prevent the progression of chronic pancreatic inflammation and fibrosis in mice [64]. In vitro studies have also demonstrated that *Pleurotus ostreatus* and *Pleurotus eryngii* polysaccharides support the growth of probiotic bacteria, particularly *Lactobacillus* [65]. Zhao et al. (2018) conducted a study to investigate the impact of six common edible fungi on the composition of fecal flora during in vitro anaerobic fermentation, in which it was found that *Flammulina velutipes*, *Lentinus edodes*, and *Pleurotus eryngii* stimulated the growth of *Actinobacteria* [66]. The results indicated that *Flammulina velutipes*, *Lentinus edodes*, and *Pleurotus eryngii* stimulated the growth of *Actinobacteria*. Additionally, the supplementation of *Agaricus bispours* was found to increase the abundance of *Actinobacteria* and *Firmicutes*, while decreasing that of *Bacteroidetes.* Additionally, *Ganoderma lucidum* polysaccharide had been found to elevate the level of *Bifidobacterium* and *Lactobacillus* at the genus level in feces [23]. These findings were consistent with our research results. They collectively suggested that polysaccharides from various sources had the potential to modulate the gut microbiota and might offer therapeutic benefits in the context of various health conditions. However, some traditionally considered probiotics, such as *Mucispirillum*, *Alloprevotella*, and pathogenic bacteria *Alistipes*, *Helicobacter* did not show the expected changes; the reason for this is currently unclear and requires further research in the future.

Beta Diversity analyzed the composition of microbial communities in different groups. PCoA analysis in Figure 7A demonstrates that the composition of microbial communities in the NC group differed significantly from that of the D-galactose induced aging group, indicating a strong association between microbial composition and aging. However, the intervention of AAPS and Bb-12 was able to partially modify this situation, suggesting that the intervention had a positive impact on the gut microbiota and potentially mitigated the effects of aging. Spearman correlation heatmap was used to investigate the relationship between changes in gut microbiota and physiological and biochemical indicators (Figure 7B). This further confirmed that AAPS and Bb-12 might delay aging by regulating the structure and abundance of the gut microbiota in aging organisms, increasing the beneficial bacteria content, and further affecting oxidative stress and inflammation associated with aging.

Although the combined use of AAPS and Bb-12 had demonstrated some progress in research on functional activities such as antioxidation and anti-aging, the molecular mechanisms of anti-aging, the target effects of drug combination, the enzymatic degradation steps and intermediate products of gut microbiota, and the nutritional symbiosis of substances with microbial interactions were still unclear. In the future, more research should focus on combining metabolomics, molecular biology, and enzymology methods to reveal the molecular mechanisms of how AAPS, combined with Bb-12, affects the body’s antioxidation and anti-aging functions.

## 5. Conclusions

This study investigated the effects of AAPS combined with Bb-12 on both *D. melanogaster* and D-galactose-induced aging mice. The findings revealed that the combination extended the lifespan of naturally aging *D. melanogaster*, improved their resistance to hydrogen peroxide oxidative stress, enhanced climbing ability, boosted antioxidant enzyme activity in both *D. melanogaster* and mice, regulated aging biomarker levels in mice, restored gut microbiota diversity and structure in mice intestines, promoted beneficial bacteria proliferation, suppressed harmful bacteria abundance, and optimized the intestinal microecological environment. These experimental results illustrated the remarkable antioxidant and anti-aging properties of AAPS combined with Bb-12, laying a theoretical groundwork for its potential use as a functional food additive and in pharmaceuticals.

## Figures and Tables

**Table 1:** Effects of AAPS and Bb-12 on the lifespan of *D. melanogaster*.

Group		Mean Lifespan (d)	Maximum Lifespan (d)	Median Survival (d)	Prolongation of Mean Lifespan (%)
Male	NC	50.72 ± 1.45	66.20 ± 0.19	55.50	-
	AA	53.83 ± 1.60	71.40 ± 0.32 **	59.50	6.13
	BB	52.14 ± 1.43	67.80 ± 0.42 **	57.50	2.80
	AB	54.99 ± 1.52 *	71.60 ± 0.47 **	61.00	8.42
Female	NC	51.59 ± 1.39	67.20 ± 0.24	55.50	-
	AA	55.61 ± 1.45 *	71.00 ± 0.32 **	62.00	7.79
	BB	53.39 ± 1.50	71.10 ± 0.33 **	59.00	3.45
	AB	56.64 ± 1.49 **	74.80 ± 0.27 **	61.00	9.79

Note: Data are presented as the means ± SEM (n = 100 per group). * *p* < 0.05 vs. NC group. ** *p* < 0.01 vs. NC group. AA, 9 mg/mL AAPS; BB, 10^9^ CFU/mL Bb-12; AB, a combination of 9 mg/mL AAPS and 10^9^ CFU/mL Bb-12; (n = 100 per group).

**Table 2:** Effects of AAPS and Bb-12 on the lifespan of H_2_O_2_ treated *D. melanogaster*.

Group		Mean Lifespan (h)	Maximum Lifespan (h)	Median Survival (h)	Prolongation of Mean Lifespan (%)
Male	NC	17.04 ± 0.51	23.10 ± 0.43	18.00	-
	AA	18.93 ± 0.59 **	25.50 ± 0.47 **	21.00	11.09
	BB	17.31 ± 0.50	24.00 ± 0.00 *	18.00	1.58
	AB	19.11 ± 0.58 **	26.40 ± 0.38 **	21.00	12.15
Female	NC	17.34 ± 0.46	24.00 ± 0.00	18.00	-
	AA	18.90 ± 0.62 *	27.30 ± 0.51 **	21.00	9.00
	BB	17.46 ± 0.40	22.80 ± 0.46 *	18.00	0.69
	AB	19.11 ± 0.61 *	27.90 ± 0.43 **	21.00	10.21

Note: Data are presented as the means ± SEM (n = 100 per group). * *p* < 0.05 vs. NC group. ** *p* < 0.01 vs. NC group.

**Table 3:** Levels of aging biomarkers and antioxidant activity of aging mice.

	NC	MC	AA	BB	AB
Aging biomarkers					
AGEs (ng/gprot)	623.44 ± 30.89	878.00 ± 125.55 ^##^	763.64 ± 84.36 ^##^	750.00 ± 127.71 ^#^	735.58 ± 86.46 *^##^
iNOS (U/mgprot)	0.56 ± 0.04	0.77 ± 0.10 ^##^	0.57 ± 0.07 **	0.60 ± 0.07 **	0.61 ± 0.06 **^#^
Telomerase (nU/mgprot)	69.61 ± 3.97	57.10 ± 6.92 ^##^	61.47 ± 4.42 ^##^	60.25 ± 1.88 ^##^	62.28 ± 5.53 ^#^
Antioxidant activity in brain					
T-SOD (U/mgprot)	351.12 ± 15.40	287.61 ± 16.00 ^##^	335.15 ± 26.44 **	333.88 ± 22.48 **	336.64 ± 20.41 **
CAT (U/mgprot)	40.73 ± 0.72	37.16 ± 1.74 ^##^	39.35 ± 0.63 *^#^	38.65 ± 1.22 ^#^	39.28 ± 1.48 *
GSH-Px (U/mgprot)	707.22 ± 31.20	619.59 ± 56.82 ^##^	688.71 ± 39.94 *	671.21 ± 33.01 ^#^	681.65 ± 46.45 *
MDA (nmol/mgprot)	13.25 ± 1.08	19.99 ± 1.93 ^##^	16.99 ± 2.25 *^##^	16.29 ± 2.64 *^#^	14.73 ± 0.83 **^#^
Antioxidant activity in serum					
T-SOD (U/mL)	82.23 ± 4.96	58.66 ± 4.28 ^##^	71.83 ± 4.80 **^##^	69.07 ± 3.02 **^##^	72.32 ± 3.55 **^##^
CAT (U/mL)	29.29 ± 1.65	22.42 ± 2.59 ^##^	25.16 ± 2.43 **^#^	25.00 ± 1.88 *^##^	27.43 ± 2.80 **
GSH-Px (U/mL)	358.41 ± 19.33	295.88 ± 33.99 ^##^	345.29 ± 21.81 **	332.13 ± 26.28 *	339.55 ± 20.00 *
MDA (nmol/mL)	4.80 ± 0.26	8.38 ± 1.61 ^##^	6.03 ± 1.29 *^#^	6.46 ± 0.54 *^##^	6.19 ± 0.67 **^##^

Note: Data are presented as the means ± SEM (n = 10 per group). * *p* < 0.05 vs. MC group, ** *p* < 0.01 vs. MC group; ^#^ *p* < 0.05 vs. NC group, ^##^ *p* < 0.01 vs. NC group.

## Data Availability

The data presented in this study are available on request from the corresponding author. The data are not publicly available due to concerns about misuse of the data.

## References

[1] López-Otín C., Blasco M.A., Partridge L., Serrano M., Kroemer G. (2013). The Hallmarks of Aging. Cell.

[2] Campisi J., Kapahi P., Lithgow G.J., Simon M., Newman J.C., Verdin E. (2019). From discoveries in ageing research to therapeutics for healthy ageing. Nature.

[3] Denham H. (1956). Aging: A theory based on free radical and radiation chemistry. J. Gerontol..

[4] Finkel T., Holbrook N.J. (2000). Oxidants, oxidative stress and the biology of ageing. Nature.

[5] Davalli P., Mitic T., Caporali A., Lauriola A., D’Arca D. (2016). ROS, cell Senescence, and novel molecular mechanisms in aging and age-related diseases. Oxidative Med. Cell. Longev..

[6] Kudryavtseva A.V., Krasnov G.S., Dmitriev A.A., Alekseev B.Y., Kardymon O.L., Sadritdinova A.F., Fedorova M.S., Pokrosky A.V., Melnikova N.V., Kaprin A.D. (2016). Mitochondrial dysfunction and oxidative stress in aging and cancer. Oncotarget.

[7] Lee K.J., Yun I.J., Kim K.H., Lim S.H., Ham H.J., Eum W.S., Joo J.H. (2011). Amino acid and fatty acid compositions of Agrocybe chaxingu, an edible mushroom. J. Food Compos. Anal..

[8] Jing H.J., Zhang Q., Liu M., Zhang J.J., Zhang C., Li S.S., Ren Z.Z., Gao Z., Liu X.T., Jia L. (2018). Polysaccharides with antioxidative and antiaging activities from enzymatic-extractable mycelium by *Agrocybe aegerita* (Brig.) Sing. J. Evid.-Based Complement. Altern. Med..

[9] Jing H.J., Li J., Zhang J.J., Wang W.S., Li S.S., Ren Z.Z., Gao Z., Song X.L., Wang X.X., Jia L. (2018). The antioxidative and anti-aging effects of acidic- and alkalic-extractable mycelium polysaccharides by *Agrocybe aegerita* (Brig.) Sing. Int. J. Biol. Macromol..

[10] Peng Y.Y., Zhang J.H., Yang H.L., Yang Z.F., Xue H.B., Wu F., Wang Z.Y.J., Xie L., Chen Y.Y. (2022). Acetylation modification and antioxidant activity of polysaccharides from *Agrocybe cylindracea*. J. Food Meas. Charact..

[11] Du B., Peng F., Xie Y., Wang H.Y., Wu J.H., Liu C., Yang Y.D. (2022). Optimization extraction and antioxidant activity of crude polysaccharide from chestnut mushroom (*Agrocybe aegerita*) by accelerated solvent extraction combined with response surface methodology (ASE-RSM). Molecules.

[12] Liu X.Y., Liu D., Chen Y.H., Zhong R.T., Gao L.Y., Yang C.F., Ai C., El-Seedi H.R., Zhao C. (2020). Physicochemical characterization of a polysaccharide from *Agrocybe aegirita* and its anti-ageing activity. Carbohydr. Polym..

[13] Liu X.Y., Wu L.X., Tong A.J., Zhen H.M., Han D., Yuan H.Y., Li F.N., Wang C.T., Fan G.S. (2022). Anti-aging effect of *Agrocybe aegerita* polysaccharide through regulation of oxidative stress and gut microbiota. Foods.

[14] Wu L.X., Liu X.Y., Hu R.K., Chen Y.X., Xiao M.F., Liu B., Zeng F. (2022). Prebiotic *Agrocybe cylindracea* crude polysaccharides combined with *Lactobacillus rhamnosus GG* postpone aging-related oxidative stress in mice. Food Funct..

[15] Ji Y., Zheng M.F., Ye S.G., Wu X.B., Chen J.Y. (2013). *Agrocybe aegerita* polysaccharide combined with chemotherapy improves tumor necrosis factor-α and interferon-γ levels in rat esophageal carcinoma. Dis. Esophagus.

[16] Lin S.L., Ching L.T., Lam K., Cheung P.C.K. (2017). Anti-angiogenic effect of water extract from the fruiting body of *Agrocybe aegerita*. LWT-Food Sci. Technol..

[17] Li G., Liu X., Cong S., Deng Y., Zheng X. (2021). A novel serine protease with anticoagulant and fibrinolytic activities from the fruiting bodies of mushroom *Agrocybe aegerita*. Int. J. Biol. Macromol..

[18] Taormina G., Ferrante F., Vieni S., Grassi N., Russo A., Mirisola M.G. (2019). Longevity: Lesson from model organisms. Genes.

[19] Weinrich T.W., Coyne A., Salt T.E., Hogg C., Jeffery G. (2017). Improving mitochondrial function significantly reduces metabolic, visual, motor and cognitive decline in aged *Drosophila melanogaster*. Neurobiol. Aging.

[20] Mantovani M.S., Bellini M.F., Angeli J.P.F., Oliveira R.J., Silva A.F., Ribeiro L.R. (2008). β-glucans in promoting health: Prevention against mutation and cancer. Mutat. Res. Rev. Mutat. Res..

[21] Kaoutari A.E., Armougom F., Gordon J.I., Raoult D., Henrissat B. (2013). The abundance and variety of carbohydrate-active enzymes in the human gut microbiota. Nat. Rev. Microbiol..

[22] Liang J.J., Zhang M.N., Wang X.N., Ren Y.C., Yue T.L., Wang Z.L., Gao Z.P. (2021). Edible fungal polysaccharides, the gut microbiota, and host health. Carbohydr. Polym..

[23] Guo C.L., Guo D.D., Fang L., Sang T.T., Wu J.J., Guo C.J., Wang Y.J., Wang Y., Chen C.J., Chen J.J. (2021). *Ganoderma lucidum* polysaccharide modulates gut microbiota and immune cell function to inhibit inflammation and tumorigenesis in colon. Carbohydr. Polym..

[24] Ling Z.X., Liu X., Cheng Y.W., Yan X.M., Wu S.C. (2022). Gut microbiota and aging. Crit. Rev. Food Sci..

[25] Jayanama K., Theou O. (2020). Effects of probiotics and prebiotics on frailty and ageing: A narrative review. Curr. Clin. Pharmacol..

[26] Chenhuichen C., Cabello-Olmo M., Barajas M., Izquierdo M., Ramirez-Velez R., Zambom-Ferraresi F., Martinez-Velilla N. (2022). Impact of probiotics and prebiotics in the modulation of the major events of the aging process: A systematic review of randomized controlled trials. Exp. Gerontol..

[27] Toward R.E., Montandon S.L., Walton G.E., Gibson G.R. (2012). Effect of prebiotics on the human gut microbiota of elderly persons. Gut Microbes.

[28] Pandey K.R., Naik S.R., Vakil B.V. (2015). Probiotics, prebiotics and synbiotics- a review. J. Food Sci. Technol.-MYSORE.

[29] Mohanty D., Misra S., Mohapatra S., Sahu P.S. (2018). Prebiotics and synbiotics: Recent concepts in nutrition. Food Biosci..

[30] Martinez V.G., Javadi C.S., Ngo E., Ngo L., Lagow R.D., Zhang B. (2007). Age-related changes in climbing behavior and neural circuit physiology in *Drosophila*. Dev. Neurobiol..

[31] Kidera H., Hatabu T., Takahashi K.H. (2020). Apoptosis inhibition mitigates aging effects in *Drosophila melanogaster*. Genetica.

[32] Iliadi K.G., Boulianne G.L. (2010). Age-related behavioral changes in *Drosophila*. Ann. N. Y. Acad. Sci..

[33] Wickens A.P. (2001). Ageing and the free radical theory. Respir. Physiol..

[34] Jyoti A., Mishra N., Dhas Y. (2016). Ageing: Consequences of excessive free radicals and inflammation. Curr. Sci..

[35] Valko M., Leibfritz D., Moncol J., Cronin M.T.D., Mazur M., Telser J. (2007). Free radicals and antioxidants in normal physiological functions and human disease. Int. J. Biochem. Cell Biol..

[36] Morava E. (2014). Galactose supplementation in phosphoglucomutase-1 deficiency; review and outlook for a novel treatable CDG. Mol. Genet. Metab..

[37] Azman K.F., Zakaria R. (2019). D-Galactose-induced accelerated aging model: An overview. Biogerontology.

[38] Haider S., Liaquat L., Shahzad S., Sadir S., Madiha S., Batool Z., Tabassum S., Saleem S., Naqvi F., Perveen T. (2015). A high dose of short term exogenous D-galactose administration in young male rats produces symptoms simulating the natural aging process. Life Sci..

[39] Shwe T., Pratchayasakul W., Chattipakorn N., Chattipakorn S.C. (2018). Role of D-galactose-induced brain aging and its potential used for therapeutic interventions. Exp. Gerontol..

[40] Poljsak B., Suput D., Milisav I. (2013). Achieving the Balance between ROS and antioxidants: When to use the synthetic antioxidants. Oxidative Med. Cell. Longev..

[41] Zhao H.Q., Zhang R.F., Yan X.Y., Fan K.L. (2021). Superoxide dismutase nanozymes: An emerging star for anti-oxidation. J. Mater. Chem. B.

[42] Morales M., Munné-Bosch S. (2019). Malondialdehyde: Facts and artifacts. Plant Physiol..

[43] Frimat M., Daroux M., Litke R., Nevière R., Tessier F.J., Boulanger E. (2017). Kidney, heart and brain: Three organs targeted by ageing and glycation. Clin. Sci..

[44] Byun K., Yoo Y., Son M., Lee J., Jeong G.B., Park Y.M., Salekdeh G.H., Lee B. (2017). Advanced glycation end-products produced systemically and by macrophages: A common contributor to inflammation and degenerative diseases. Pharmacol. Ther..

[45] Zvereva M.I., Shcherbakova D.M., Dontsova O.A. (2010). Telomerase: Structure, functions, and activity regulation. Biochemistry.

[46] Biagi E., Franceschi C., Rampelli S., Severgnini M., Ostan R., Turroni S., Consolandi C., Quercia S., Scurti M., Monti D. (2016). Gut microbiota and extreme longevity. Curr. Biol..

[47] Tuikhar N., Keisam S., Labala R.K., Imrat, Ramakrishnan P., Arunkumar M.C., Ahmed G., Biagi E., Jeyaram K. (2019). Comparative analysis of the gut microbiota in centenarians and young adults shows a common signature across genotypically non-related populations. Mech. Ageing Dev..

[48] Park S.H., Kim K.A., Ahn Y.T., Jeong J.J., Huh C.S., Kim D.H. (2015). Comparative analysis of gut microbiota in elderly people of urbanized towns and longevity villages. BMC Microbiol..

[49] Kumar M., Babaei P., Ji B., Nielsen J. (2015). Human gut microbiota and healthy aging: Recent developments and future prospective. Nutr. Healthy Aging.

[50] Mariat D., Firmesse O., Levenez F., Guimaraes V.D., Sokol H., Doré J., Corthier G., Furet J.P. (2009). The *Firmicutes*/*Bacteroidetes* ratio of the human microbiota changes with age. BMC Microbiol..

[51] Ghosh T.S., Arnoux J., O’Toole P.W. (2020). Metagenomic analysis reveals distinct patterns of gut lactobacillus prevalence, abundance, and geographical variation in health and disease. Gut Microbes.

[52] Rastogi S., Singh A. (2022). Gut microbiome and human health: Exploring how the probiotic genus *Lactobacillus* modulate immune responses. Front. Pharmacol..

[53] Huang Y.G., Qi H.B., Zhang Z.Q., Wang E.L., Yun H., Yan H., Su X.M., Liu Y.Q., Tang Z.Z., Gao Y.H. (2017). Gut REG3γ-associated *Lactobacillus* induces anti-inflammatory macrophages to maintain adipose tissue homeostasis. Front. Immunol..

[54] Won S.M., Lee N.Y., Oh K.K., Gupta H., Sharma S.P., Kim K.H., Kim B.K., Joung H.C., Jeong J.J., Ganesan R. (2023). Gut *Lactobacillus* and Probiotics *Lactobacillus lactis/rhamnosis* ameliorate liver fibrosis in prevention and treatment. J. Microbiol..

[55] Sanborn V., Azcarate-Peril M.A., Updegraff J., Manderino L., Gunstad J. (2020). Randomized clinical trial examining the impact of *Lactobacillus rhamnosus* GG probiotic supplementation on cognitive functioning in middle-aged and older adults. Neuropsychiatr. Dis. Treat..

[56] Kong Y.Z., Olejar K.J., On S.L.W., Chelikani V. (2020). The potential of *Lactobacillus* spp. for modulating oxidative stress in the gastrointestinal tract. Antioxidants.

[57] Finamore A., Ambra R., Nobili F., Garaguso I., Raguzzini A., Serafini M. (2018). Redox role of *Lactobacillus casei Shirota* against the cellular damage induced by 2,2 -azobis (2-amidinopropane) dihydrochloride-induced oxidative and inflammatory stress in enterocytes-like epithelial cells. Front. Immunol..

[58] Li B.L., Evivie S.E., Lu J.J., Jiao Y.H., Wang C.F., Li Z.Y., Liu F., Huo G.C. (2018). *Lactobacillus helveticus* KLDS1.8701 alleviates d-galactose-induced aging by regulating Nrf-2 and gut microbiota in mice. Food Funct..

[59] Liu R.L., Qiao Y., Huang G.Q., Qian W., Xiong J.L., Wang X.Y., Zheng W.J., Yao W. (2022). Effect of synbiotic containing *Bacillus coagulans* and *Lactulose* on gut health in mice with DSS-induced ulcerative colitis. Acta Microbiol. Sin..

[60] del Campo F.M., Magaña N.V., Salazar-Félix N.A., Rodríguez M.P., Romo-Flores M.D., Sanabria L.C., Campos E.R., Manzano A.M.C. (2020). *Odoribacter* and *Anaerotruncus*: Gut microbiome signature might be related cognitive impairment in patients on peritoneal dialysis. Nephrol. Dial. Transplant..

[61] Hamilton A.L., Kamm M.A., Ng S.C., Morrison M. (2018). *Proteus* spp. as putative gastrointestinal pathogens. Clin. Microbiol. Rev..

[62] Chen J., Liu J.J., Yan C.C., Zhang C., Pan W.J., Zhang W.N., Lu Y.M., Chen L., Chen Y. (2020). *Sarcodon aspratus* polysaccharides ameliorated obesity-induced metabolic disorders and modulated gut microbiota dysbiosis in mice fed a high-fat diet. Food Funct..

[63] Chen M.Y., Xiao D., Liu W., Song Y.F., Zou B.R., Li L., Li P., Cai Y., Liu D.L., Liao Q.F. (2020). Intake of *Ganoderma lucidum* polysaccharides reverses the disturbed gut microbiota and metabolism in type 2 diabetic rats. Int. J. Biol. Macromol..

[64] Ren G.M., Yu M., Li K.K., Hu Y., Wang Y., Xu X.H., Qu J.J. (2016). Seleno-lentinan prevents chronic pancreatitis development and modulates gut microbiota in mice. J. Funct. Foods.

[65] Synytsya A., Mícková K., Synytsya A., Jablonsky I., Spevácek J., Erban V., Kováríková E., Copíková J. (2009). Glucans from fruit bodies of cultivated mushrooms *Pleurotus ostreatus* and *Pleurotus eryngii*: Structure and potential prebiotic activity. Carbohydr. Polym..

[66] Zhao R.Q., Yang W.J., Pei F., Zhao L.Y., Hu Q.H. (2018). In vitro fermentation of six kinds of edible mushrooms and its effects on fecal microbiota composition. LWT—Food Sci. Technol..

